# Neural Correlates of Freezing of Gait in Parkinson's Disease: An Electrophysiology Mini-Review

**DOI:** 10.3389/fneur.2020.571086

**Published:** 2020-11-10

**Authors:** J. Sebastian Marquez, S. M. Shafiul Hasan, Masudur R. Siddiquee, Corneliu C. Luca, Virendra R. Mishra, Zoltan Mari, Ou Bai

**Affiliations:** ^1^Department of Electrical and Computer Engineering, Florida International University, Miami, FL, United States; ^2^Department of Neurology, University of Miami Hospital, Miami, FL, United States; ^3^Lou Ruvo Center for Brain Health, Cleveland Clinic, Las Vegas, NV, United States

**Keywords:** Parkinson's disease (PD), freezing of gait (FoG), electrophysiology, cortical, subcortical

## Abstract

Freezing of gait (FoG) is a disabling symptom characterized as a brief inability to step or by short steps, which occurs when initiating gait or while turning, affecting over half the population with advanced Parkinson's disease (PD). Several non-competing hypotheses have been proposed to explain the pathophysiology and mechanism behind FoG. Yet, due to the complexity of FoG and the lack of a complete understanding of its mechanism, no clear consensus has been reached on the best treatment options. Moreover, most studies that aim to explore neural biomarkers of FoG have been limited to semi-static or imagined paradigms. One of the biggest unmet needs in the field is the identification of reliable biomarkers that can be construed from real walking scenarios to guide better treatments and validate medical and therapeutic interventions. Advances in neural electrophysiology exploration, including EEG and DBS, will allow for pathophysiology research on more real-to-life scenarios for better FoG biomarker identification and validation. The major aim of this review is to highlight the most up-to-date studies that explain the mechanisms underlying FoG through electrophysiology explorations. The latest methodological approaches used in the neurophysiological study of FoG are summarized, and potential future research directions are discussed.

## Introduction

Freezing of gait (FoG) affects over half the population with advanced Parkinson's disease (PD) (1). This highly disabling symptom is defined as “brief episodes of inability to step or by extremely short steps that typically occur on initiating gait or on turning while walking” (2). FoG is often responsible for falls within PD and is highly associated with recurrent falls (3, 4). Although there are pharmacological, therapeutic, and surgical treatments to alleviate PD-FoG, evidence to support their effects is inconclusive (5). Understanding what causes FoG has a tremendous public health impact because FoG is the leading cause of disabling falls. To date, no accepted model completely explains FoG as this symptom is complex, is difficult to capture under observation, and varies during manifestation.

FoG can be provoked by different triggers including turning, multi-tasking, and emotional states (6). Due to the nature of FoG and its multi-systemic neural impairments, disturbances to individual brain regions are difficult to isolate. This further complicates the understanding of the symptom, which causes medication intervention and treatment to be unfocused, non-specific, and unsuccessful. To this effect, many research groups have used a variety of methods and approaches to understand the pathophysiological mechanisms behind FoG. Among the most notable approaches are behavioral and clinical measures ranging from gait and posture (7–13) to upper limb biomechanics (14), jaw displacement (15), saccade latency, velocity, and gain (16), audio-spinal reflex (17), handwriting measures (18), foot pedal measures (19), speech (20), electromyography (EMG) (21, 22), and even rapid eye movement frequency (23). However, to explore the physiological changes specifically related to FoG in neurological pathways and their effect on motor control, it is necessary to detect efficacious neural biomarkers that may differentiate PD from healthy controls and PD subtypes from one another. Great efforts have been made to study neural features that differentiate PD patients who exhibit FoG (PDFoG+) vs. those who do not (PDFoG–). To accomplish this, the research tools that have been applied include functional near-infrared spectroscopy (fNIRS) (24), electroencephalography (EEG) (13, 25–28), deep brain electrode-based recordings (26, 29–33), and various neuroimaging tools such as volumetric magnetic resonance imaging (vMRI), diffusion MRI (dMRI), functional MRI (fMRI) (34–37), and positron emission tomography (PET) (35, 36). It is well-known that each of these methods has advantages over the others, for example, the spatial vs. temporal resolution advantage held by fMRI over EEG, or the non-invasive vs. surgical advantage of EEG over deep brain electrode-based recordings. The main aim of this review is to summarize the latest studies on mechanism theories underlying FoG in PD from an electrophysiological scope. The approaches of past studies are discussed to highlight their importance in neural biomarker exploration.

## Pathophysiology Mechanisms Underlying FoG

Locomotion is a complex process that involves automatic, emotional, and volitional control (38–40). In non-automatic gait, the initiation command is generated at the cerebral cortex and is executed by the thalamocortical, corticobulbar, and spinal projections networks (38). Automatic rhythmicity, posture preparations, and adjustments during locomotion are regulated by the brainstem and spinal cord after gait is volitionally initiated. Meanwhile, the cerebellum simultaneously takes in the sensory feedback from the spinal cord and feed-forward information from the cortex to regulate predictive control (41). In PD, multilevel network failure may ultimately lead to FoG events. However, the neural complexity leads to uncertainty in identifying and isolating specific neural impairments that result in FoG. To explain the pathophysiology, five non-exclusive mechanisms have been suggested regarding the physiological alterations leading to FoG (2, 6).

**Figure 1:**
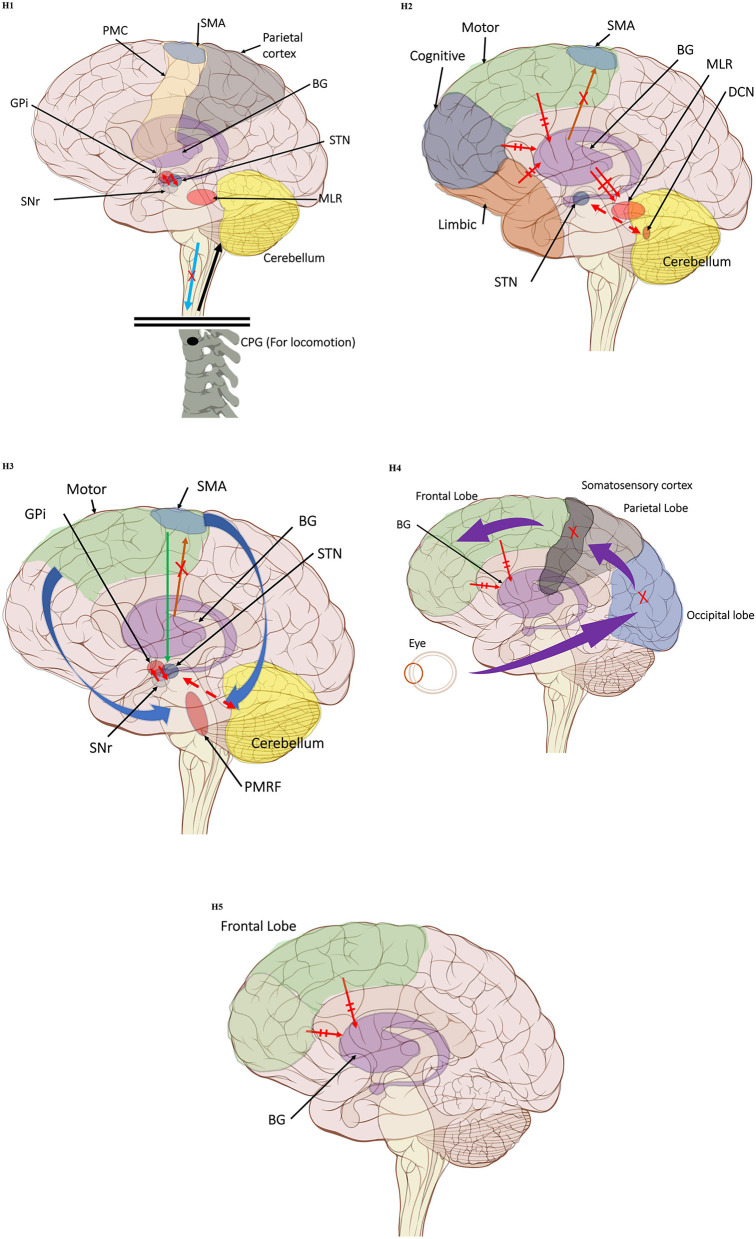
(H1) Abnormal control outputs from central pattern generators. The supraspinal locomotor network (SLN) is composed of the premotor cortex (PMC), supplementary motor area (SMA), parietal cortex, basal ganglia (BG), subthalamic nucleus (STN), mesencephalic locomotor region (MLR), and cerebellum. 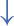 In normal function, the SLN network sends cues for turning, stopping, obstacle maneuvering, and new locomotion goals. X In PD, FoG is caused by the disruption of SLN cues. 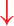 Disruption is caused by inhibition of the globus pallidus internus (GPi)/substantia nigra pars reticulata (SNr) pathway, resulting in decreased rhythmic control. (H2) A disconnect between the basal ganglia and the supplementary motor area or crosstalk to basal ganglia. 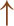 In normal function, BG-SMA sends internal cueing for automatic initiation of previously learned tasks. X In PD, FoG is caused by the disruption of BG-SMA cues. This disconnect leads to the inability to multitask. 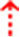 The increased inhibitory output from deep cerebellar nuclei (DCN) further prevents the execution of habitual responses. 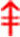 In PD, crosstalk between the input to BG from the cognitive, motor, and limbic cortices may also trigger FoG. 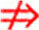 Firing in the output nuclei of the BG inhibits MLR, leading to FoG events. (H3) Knee-trembling and the abnormal coupling between posture and gait. 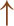 In normal function, BG-SMA sends internal cueing for the automatic initiation of previously learned tasks. Because of the dopamine depletion, executive function is lost. X The BG then fails to convey appropriate anticipatory postural adjustments. 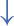 The breakdown of coupling between posture preparation (SMA) and step initiation (motor cortex) might occur at the pontomedullary reticular formation (PMRF), which plays a role in postural control and regular locomotion. 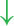 The hyperdirect pathway (SMA-STN) becomes engaged as a result of the coupling breakdown activating the GPi/SNr pathway. 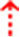 This additionally affects cerebellar automatic gait processing. 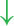 GPi/SNr oscillations may underpin characteristic 3–8 Hz knee-trembling. (H4) A perceptual malfunction and slowing down when passing doorways. 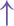 In normal function, the dorsal stream takes visual information to the occipitoparietal stream, where somatosensory signals are then transferred to the frontal lobe for the origination of motor function intent. X In PD, FoG events are caused by dysfunctional dorsal stream processing, which consequently causes inappropriate adaptation of locomotion. (H5) A consequence of frontal executive dysfunction. 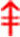 In PD, FoG events are caused by a disconnect between the frontal lobe and the BG. This disconnect results in poor performance of multiple consequent tasks and the characteristically low frontal assessment battery and verbal fluency scores. Highlighted regions and added text and arrows to Medical Illustrations by Patrick Lynch, generated for multimedia teaching projects by the Yale University School of Medicine, Center for Advanced Instructional Media, 1987-2000. Patrick J. Lynch, http://patricklynch.net Creative Commons.

The first hypothesis states that the characteristic gait features associated with FoG are caused by abnormal control outputs from the central pattern generators (CPG) in the spinal cord (9) (Figure 1 H1). The CPG is a neural network in charge of goal-directed motor output independent of external timing cues or sensory feedback (38). In addition to rhythmic control, the CPG also receives commands from the supraspinal locomotor network, which is made up of the primary motor cortex, the supplementary motor cortex, the parietal cortex, the BG, the subthalamic nucleus, the mesencephalic locomotor region, and the cerebellum (39). In PD, FoG events may be caused by a disruption of the supraspinal control cues to the CPG, which are crucial for turning, stopping, maneuvering obstacles, and adapting to new locomotion goals (2). This explanation takes into consideration the fact that, in the control pathway of healthy individuals, the sensorimotor striatum that is in charge of habitual behavior (40) is inhibited by the substantia nigra pars compacta (SNc) through D2 dopamine receptors, which are involved in locomotion, learning, memory, and reproductive behavior (41). During normal operation, inhibition of the striatum results in inhibition of the globus pallidus pars externus (GPe). The active GPe then allows for inhibition of the globus pallidus internus/substantia nigra pars reticulata pathway (GPi/SNr), resulting in the execution of previously learned tasks (42). In the case of PD, the lack of synergy between dopaminergic modulation networks leads to inhibitions of the GPi/SNr pathway, resulting in decreased rhythmic gait control (2, 42–45). Although not specifically the focus of this review, it is worth noting that the formerly popular direct/indirect pathway's simplistic assumptions regarding loops and purely excitatory/inhibitory connections have been questioned and debunked to a significant extent (46).

The second mechanistic hypothesis underlying the pathophysiology of FoG states that, due to the disconnect from the BG to the supplementary motor area (SMA), the internal cueing that automatically initiates previously learned movements is interrupted (47) (Figure 1 H2). Some studies considered that FoG might be due to the loss of automatic updating of motor programs by the dysfunctional BG in PD (43, 48), leading to the inability to multitask (2, 44). Furthermore, because of the increased competition between the excitatory output from the subthalamic nucleus (STN), increased inhibitory output on the deep cerebellar nuclei hinders the ability to rely on previously learned habitual responses (2, 45). Moreover, FoG might be triggered by crosstalk of complementary, yet competing, BG inputs from motor, cognitive, and limbic cortical areas (2). In this model, the synchronous firing in the output nuclei of the BG leads to increased inhibition in brainstem locomotor areas and consequently to FoG events (45). The associative striatum and frontal-parietal cortices are relatively spared early in the disease course, allowing PDFoG+ to operate gait through goal-directed strategies. However, as a result, gait becomes less automated and vulnerable to interference from concurrent task demands that can disrupt gait control (44).

From the observation of the characteristic knee-trembling during FoG, the third hypothesis states an abnormal coupling between posture and gait, which leads to multiple anticipatory postural adjustments (APA) preceding FoG events (2, 49, 50) (Figure 1 H3). This finding suggests that the inability to couple normal APAs with the stepping motor pattern is what causes FoG events in PD during a forward imbalance. APA is needed for normal gait initiation, resulting in a frozen state of attempted readiness (51). Breakdown in the coupling between posture preparation by the SMA and step initiation by the motor cortex might occur in the pontomedullary reticular formation (2, 49). The hyperdirect pathway likely becomes engaged as a result of increased response conflict, activating the GPi/SNr while also disrupting cerebellar processing involved with automated gait modulation. Altered 3–8 Hz oscillations between the STN and GPi may also underpin the characteristic “trembling in place” often observed during FoG (6, 52).

The fourth hypothesis states that the FoG event is caused by a perceptual malfunction, which in association with online planning causes PDFoG+ to decrease speed in response to the visual input related to locomotion planning (53, 54) (Figure 1 H4). Almeida and Lebold found decreased step length and increased gait variability while approaching a narrow doorway for PDFoG+ (55, 56). However, the previous study showed that PDFoG+ do not overestimate door widths more than PDFoG–; rather, they misjudge the speed reduction resulting from passing through doorways (57). The inappropriate activation of locomotor adaptation is considered to be the result of dysfunctional dorsal stream processing (58), which helps with spatial location and guides movement response (59).

The fifth hypothesis states that FoG is a consequence of frontal executive dysfunction, where there is a disconnect between the BG and the frontal lobe, resulting in poor performance when trying to achieve multiple tasks (Figure 1 H5). This is further supported by low scores in frontal assessment battery scores and verbal fluency in PDFoG+ compared to PDFoG– (6, 52, 60). Extra-nigral pathology impairs compensatory attentional gait strategies and contributes to L-dopa-resistant FoG, especially with PD progression (44).

The various hypotheses are a testament to the complexity of the underlying mechanisms of FoG, likely the heterogeneity of contributing factors among patients. Therefore, continued exploration of the pathophysiology of FoG in PD is needed. Moreover, not all PD who exhibit FoG events are triggered by the same causes or respond equally to medication or therapy. Those who may only experience events when not under observation make FoG evaluation and understanding in the clinic elusive (6). This review concentrates on examining the previous works that relied on electrophysiology to analyze neural biomarkers associated with FoG. For neuroimaging studies delving into PD-FoG, the reader is encouraged to refer to recent review papers (36, 37).

## Literature and Research Methods

Following the Preferred Reporting Items for Systematic Reviews and Meta-Analyses statement (61), the eligibility criteria for articles included those published before March 2020, written in English, and currently published journal or full-length conference articles. PubMed was used as the primary information source with the search query “EEG OR LFP AND Parkinson's Disease AND Freezing of Gait.” The search query was conducted by two independent authors who then combined the findings. This search yielded a total of 34 articles, 10 of which were removed. Six were removed because the terms “Freezing of Gait,” “EEG,” or “Parkinson's Disease” were used in the introduction but not as part of the research objective (14, 62–66). The other four were removed because the articles did not focus on electrophysiology for its methods (24, 67), because the article was either a poster abstract and not yet a full publication (68), or because the article did not deal with human subjects (66). To complement the neurophysiology review aspect of this paper, in addition to the articles found through the PubMed search, sources such as Scopus or Google Scholar were used when clarification was needed.

## Neurophysiology Studies of FoG

Recent advances in wearable electronics and computing have allowed for the miniaturization of electronics and the transition from bulky instrumentation to wearable and non-intrusive tools. This shift has allowed for neural biomarker explorations in environments that closely match everyday activities (69) and even allowed for the implementation of protocols during states of high activity, such as walking (70, 71) and cycling (72, 73). Such daily activity paradigms allow for more precise interpretation of results because parallel functions in studies using motor imagery (74) or treadmill walking (63) have to be considered. Furthermore, the constraints of simplified tasks of finger/wrist flexion to avoid motion artifact (75, 76) are no longer a big concern (44, 77, 78). Although some of the studies resulting from the PubMed query did not rely on wearable systems for their instrumentation, their findings can be translated into current and future studies using such wearable technology. This review begins with the cortical studies that use EEG to explore neural biomarkers and/or behavioral measures, then moves more deeply into subcortical studies while discussing the relevant cortical and subcortical loop connections.

### Cortical Level

Locomotion is a complex process involving gait initiation at the cerebral cortex and automatic rhythmic execution regulated by the brainstem and spinal cord. Another important contributing factor is the simultaneous predictive control through feed-forward processing by the cerebellum. These intricate mechanisms may account for the lack of consensus in FoG pathophysiology, given that exploring the complete projection system results in an overwhelming analytical task. The difficulty with selectively studying individual networks or components arises because these networks are all engaged inter-relatedly during real-life locomotion. In the case of EEG, pyramidal cells account for most of the scalp-recorded electrical signals. This non-invasive tool is thus frequently used in the exploration of the cerebral cortex, which is shown as the processing center for proprioception, predictive operations (79), and APA (80, 81). Of the 24 resulting studies from the search query, 16 studies used EEG in their approach to detect or predict FoG events or study neural changes with FoG.

For FoG biomarker exploration, several studies have focused on the study of the cortical activity associated with self-paced upper limb motion in EEG (75, 82–85). A negative potential, which starts 1–2 s before the limb movement, has been found in EEG electrodes over the central area, namely the readiness potential or Bereitschaftspotential (BP). The BP is a part of the slow cortical movement–related potentials and is followed by the pre-motor positivity (86). Early BP, which occurs 1–2 s before limb movement, is associated with activation from the pre-supplementary motor area (pre-SMA), SMA, and Brodmann area 6 (86). The late BP that occurs 400–500 ms before limb movement is associated with activation of the primary motor cortex (87). Studies have demonstrated that the amplitude of early BP is reduced in persons with PD compared to healthy controls (84, 88, 89). In contrast to the amplitude reduction in the early BP, late BP amplitude is increased in PD compared with healthy persons. The increased BP amplitude may indicate a compensation mechanism used to achieve limb control in the absence of information handover from the BG to the SMA (84). These results have been largely reproduced only in persons with PD while performing upper limb tasks (86), due largely to motion artifacts abundant with movements of other parts of the body. In addition to BP, Butler et al. (28) investigated the lateralized readiness potential (LRP) associated with cued response to the target detection tasks. They found a significantly earlier onset and a larger amplitude of the LRP in PDFoG+ compared to PDFoG–. This indicates excessive recruitment of lateral premotor areas due to the loss of automatic motor control. Their finding is in agreement with the second mechanism hypothesis, suggesting that deficit in attentional set-shifting is caused by loss of automatic updating of motor programs due to the dysfunctional BG-SMA pathway (2, 28, 44).

Previous studies related to lower limb control have mainly investigated the difference between participants with PD and healthy controls (90). Relations between stride-length changes and movement-related cortical potential (MRCP) have also been explored in PDFoG+ and PDFoG– (91). The study by Shoushtarian et al. (91) found that a reduction in the early slope of the MRCP was coupled with stride-length reduction for PDFoG– but not for PDFoG+. Their findings show electrical evidence that cortical disturbances correlate with stride-length reduction in PDFoG–, whereas the PDFoG+ showed no stride-length relationship (91). However, this study has several experimental design limitations, including the short gait cycle duration, consisting of only three steps, which is not enough to guarantee gait programming is generated by automation rather than attentional strategies. Similar studies on neural signatures of FoG have focused on evaluating the cortico-muscular relationship between the healthy elder and PDFoG+/PDFoG– (92, 93). Among them, Gunther et al. (93) found a pronounced relationship between EMG and EEG at the beginning of FoG events and voluntary stops. However, this finding was not clearly discussed in the context of its physiological explanation, and there was no significant difference between the voluntary stops and FoG events (93).

In addition to the MRCPs, time-frequency analysis is another method for evaluating neural patterns. This approach involves the evaluation of frequency bands (25, 94), event-related synchronization (ERS), and desynchronizations (ERD) (95), as well as information flow to examine how a physical manifestation can be correlated to a neural occurrence. In the work by Singh et al., theta and beta bands were evaluated during lower-limb pedaling in PDFoG+ and PDFoG– (96). Results from this study showed that compared to PDFoG–, PDFoG+ exhibited an attenuated mid-frontal theta (4–8 Hz) power, which is associated with impairments in cognitive control (97, 98). An increased beta (13–30 Hz) power was also found in PDFoG+ compared to PDFoG–. Because beta-band oscillations are considered a result of interactions between the frontal region and the BG (26, 99, 100), an increased beta suggests an increased coupling between the frontal region and the BG in PDFoG+ compared to PDFoG–. Moreover, in the study of electrophysiological signature during actual FoG events, Shine et al. (25) found an increase in theta-band power during FoG episodes within the central and frontal areas (25). These results suggest that FoG is related to the frontoparietal processing of conflict-related signals (35, 101, 102). This study also observed an increase in beta activity over the parietal area during the transition from normal walking to FoG events. The increased beta activity indicated that the frontal generated motor plans failed to reach the motor cortex, resulting in the FoG events (103). Similar STN coherence findings suggest a relation to parkinsonian limb tremor frequencies (2–10 Hz) in deep brain recording studies (104). Further works by Toledo et al. (26) during seated position also found greater high-beta (21–35 Hz) activity during the OFF dopaminergic medication, in the STN for PDFoG+ *vs*. PDFoG– (26). During the ON dopaminergic state, the high-beta power in PDFoG+ was reduced in addition to FoG event cessation. Furthermore, coherence was found between the low-beta component (at C3 and C4) as well as the high-beta coherence (between the STN and the SMA at Cz). Altogether, this study indicated that FoG events are caused by a malfunction of the frontal cortex-BG networks via the motor and associative STN loops (26).

In addition to the studies of neural correlates between EEG oscillations and lower limb motor execution, Tard et al. (105) explored motor preparation preceding motor execution, which is altered in PDFoG+ (105). In this study, an oddball discrimination task was used to compare the ERD and ERS of PDFoG+, PDFoG–, and age-matched healthy controls. Results showed that even though all groups discriminated the target from the random stimuli, as shown by the event-related potential (ERP) following the auditory preparatory stimuli, no significant beta ERD was observed in PDFoG+ compared to the other two groups. This indicated that although the PDFoG+ correctly perceived the stimulus, their post-perceptual and integrative processing might have been impaired (26), suggesting a physiological dysfunction between the frontal cortex and the BG within the motor and associative STN loops. The BG alterations were related to either hypersynchronized thalamocortical activity or reduced BG involvement (106, 107). Additionally, the longer ERS in PDFoG+ is a marker of the high power beta oscillation in the STN, which results from the cortico-subthalamic synchronization at the midline area (108). In a visual feedback study, Velu et al. (109) studied the cortical information flow between the occipital (Oz), parietal (P4), and motor (Cz) networks between six healthy controls and two PDFoG+ [one who was responsive to the visual feedback (PDr) and one who was not (PDnr)]. This study found a decrease in the beta-band power in PDr when the visual cues were presented. Furthermore, the PDr showed an increased beta-band information flow from the visual area to the motor area and from the visual area to the parietal area, as well as a decrease in the power of delta and alpha bands. The beta-band power decrease and information flow findings suggest a correlation between the visual cue and movement initiation (109).

### Subcortical Level

Utilizing EEG for gait exploration is a non-invasive way of analyzing the initial phases of locomotion, which are generated at the cerebral cortex. However, after the gait is initiated, thalamocortical, cortico-brainstem, and spinal projection produce motor programs that result in rhythmic gait control (81). Additionally, both the pedunculopontine nucleus (PPN) (33, 81, 110) and STN (26, 29, 31), which are located deep in the brainstem, have been considered to be largely involved in gait failure for persons with PD. Of the 24 studies from the search query, 8 used subcortical recordings to either study the deep brain stimulation (DBS) frequencies that resulted in the most FoG reduction (111) or evaluate frequency band changes during the seated state (26), stepping as a walking surrogate state (31, 33), bicycling state (32, 73), or during an active walking state (29, 30).

Anidi et al. (111) investigated whether beta oscillations can be used to differentiate PD FoG phenotypes under no DBS, DBS at 60 Hz, and DBS at 140 Hz from eight PDFoG+ and four PDFoG–. They observed that the beta burst durations were significantly longer in PDFoG+ compared to PDFoG– when walking in place or forward walking. Additionally, for PDFoG+ during stepping while freezing, beta bursts were longer than during stepping without freezing. Both 60- and 140-Hz DBS stimulation resulted in improved gait arrhythmicity in freezers compared to no stimulation. During stepping in place, 60- and 120-Hz stimulation attenuated beta burst power. Though 60-Hz stimulation had a greater effect in reducing the beta burst power, both stimulation frequencies attenuated the beta burst power during forward walking (111).

Toledo et al. (26) reported a relationship between STN activity and EEG frequency coherence while subjects were sitting. During the OFF state with deprivation of levodopa administration, PDFoG+ showed greater high-beta (21–35 Hz) activity in the STN compared to PDFoG– (26). During the ON state with levodopa administration, similar high-beta levels were found in both the PDFoG+ and PDFoG– groups. They considered that the high-beta power reduction may have been mediated by levodopa administration, which also suppressed the FoG occurrence. Altogether, this study provided a solid basis to support the pathophysiological mechanism leading to FoG, indicating that FoG events are caused by a malfunction of the frontal cortex-BG networks via the motor and associative STN loops (26).

In experiments where movement is restricted due to the recording setup or the movement artifacts contamination (31), stepping in place is used as a surrogate for actual gait, because stepping involves both balance control and rhythmic activity similar to the actual gait. Moreover, stepping in place is even susceptible to FoG events (11, 112). The following two studies implemented stepping-in-place paradigms as surrogates for active gait, one focusing on the changes to the PPN (33) and the other on changes to the STN (31). Fraix et al. (33) explored the oscillatory changes of the PPN in seven PDFoG+ with DBS. Their study included DBS recordings of bilateral PPN and cortical needle EEG at central and frontal areas. Their main finding was increased alpha (5–12 Hz) oscillations during stepping in place while ON levodopa compared with OFF levodopa. Contrary to other studies, they reported a beta power decrease while ON levodopa compared with OFF levodopa. This discrepancy was attributed to differences in the experimental setup as well as the long duration of STN DBS stimulation, which could have led to plastic changes in the PPN. This study also reported decreased gamma activity in the PPN while ON levodopa. This decreased gamma band activity was associated with increased PPN-cortical coherence in the gamma band. These findings supported increased phase locking between the cortex and the PPN in the gamma band, which is in agreement with an MRI study showing a direct pathway between the PPN and the frontal cortex (33, 113). Fischer et al. (31) investigated neural dynamics in the STN in persons with PD while subjects were seated and stepping in place. Their main findings include a suppression in the beta band (20–30 Hz) after ipsilateral heel strikes, which was distinct between the left and right STN sites when alternating stepping cycles. Furthermore, this study found that the auditory cueing resulted in an increased beta modulation and more synchronized gait regulation. The increased beta modulation leads to the consideration that alternate left-right DBS may redistribute beta bursts similarly to auditory cueing (31).

Bicycling is a novel experimental paradigm used to elicit movement-related neural responses with less movement artifact contaminated compared to actual gait. Research has shown that PDFoG+ may bicycle regardless of their severe walking alterations (72). Recent findings also show that cycling even promotes acute and sustained symptom improvement (114). Bicycling was associated with a stronger sustained cortical activation as indicated by high-beta power decrease. Meanwhile, cortical motor demand was decreased during sustained cycling (32). In the works by Gratkowski and Storzer, a decreased beta-band activity was observed in the STN from DBS leads. A similar beta decrease was also detected using scalp EEG from parietal and occipital areas (73). Later, Storzer et al. (115) observed a reduction of beta power in the STN during cycling compared to walking in both PDFoG+ and PDFoG– groups during the OFF state. Additionally, an 18-Hz power increase was observed in PDFoG+. The low-beta band activity was considered to be either a signature of the affected BG or a movement-inhibiting signal in the motor network (115).

Lastly, as FoG is an event that is mostly triggered during actual walking, acquiring subcortical neural biomarkers during active gait paradigms is paramount. During their active gait paradigm, Syrkin-Nikolau et al. (30) compared stepping in place, forward walking, and instrumented walking, which included a turning and barrier course. During stepping in place, a decreased STN beta-band power was found in the PDFoG+ compared to the PDFoG–. Additionally, an increased beta sample entropy, which is a measure of unpredictability, was found in PDFoG+ compared to the PDFoG–. During FoG events, increased alpha sample entropy and decreased beta power were found compared to walking without FoG. The increased sample entropy in the PDFoG+ group indicated an increase in the error of the processing of sensorimotor information in the subthalamic outflow. On the other hand, the pattern of low-beta power and high-beta entropy may be caused by a compensatory mechanism adopted to overcome abnormal gait (30). In addition to cued active gait paradigms, dual-task protocols have also been utilized to explore subcortical biomarkers of FoG. In their work, Chen et al. (29) recorded local field potentials (LFP) from the STN while subjects performed either single- or dual-task walking. In this study, FoG vulnerability was derived from accelerometers located at the subjects' trunks. Their results showed an increased LFP power in the low-beta and theta bands irrespective of single- or dual-task walking. The increased power in the low-beta and theta bands was found to be associated with FoG vulnerability. Their findings further support the second mechanism hypothesis, stating that crosstalk between motor, cognitive, and limbic circuits results in synchronous firing from the BG, resulting in FoG events (29, 45).

While electrophysiological studies provide an excellent route to capture dynamic loss in cortical connectivity with freezing in PD, electrical measures provide a limited understanding of the cortical-subcortical connectivity and whether there are homogeneous losses in brain structure within PDFoG+. PET and MRI provide the means to study the loss of brain structure in PDFoG+. These neuroimaging techniques have been extensively utilized to understand the pathophysiological mechanisms of PDFoG+ (36, 37, 116). Converging evidence from these neuroimaging techniques suggests that subthalamic locomotor regions, cerebellar locomotor regions, and mesencephalic locomotor regions may be involved with FoG. This review is focused on the electrophysiological findings related to PDFOG+, in which the studies of imaging findings are excluded. However, excellent reviews on imaging technology can be found in recent papers (36, 37, 116).

## Summary of Findings

### Differences in EEG Between PDFoG+ and PDFOG–

Table 1 summarizes the studies that focused on differentiating PDFoG+ vs. PDFoG– for developing potential biomarkers. For PDFoG+ the LRP in EEG was shown to have an earlier onset and a larger magnitude during cognitive decision-making of target detection compared to PDFoG–. These findings support the second mechanistic hypothesis, which states that dual-task-triggered FoG events are caused by the loss of automatic updating of motor programs by the dysfunctional BG (28). In terms of EEG frequency band analysis, the beta band was shown to increase in the frontal region during lower limb pedaling in PDFoG+, indicating preparatory adjustments and motor plan execution through top-down signaling (118). Also, for PDFoG+ theta band power was attenuated in the mid-frontal location, indicating an impairment in cognitive control (96). In the study that used the dual-task oddball paradigm, although the stimulus was detected by an increased P300, beta ERD was unchanged for PDFoG+. The inability of PDFoG+ to react to stimuli might have been caused by impairments at the BG. The BG impairments were suggested to be caused either by reduced input to the sensory-motor cortex or by the hypersynchronized thalamocortical activity. Additionally, the duration of beta ERS was found to be longer after the cue signal in the PDFoG+ compared with PDFoG– (105).

**Table 1:** What differentiates PD with and without FoG?

**Title**	**Protocol**	**Metric**	**Findings**	**Interpretation (Hypothesis Supported)**
Motor preparation rather than decision-making differentiates Parkinson's disease patients with and without freezing of gait (28)	Detection of differences in cognitive decision making by EEG recording during timed response target detection	Lateralized readiness potential (LRP)	Earlier onset in PDFoG+ compared with PDFoG–	Excessive recruitment of lateral premotor areas due to loss of automatic motor control (2nd)
		LRP	Larger magnitude in PDFoG+ compared with PDFoG–	
Frontal theta and beta oscillations during lower-limb movement in Parkinson's disease (117)	EEG recording during lower-limb pedaling	Theta (4–8) power	Attenuated in the mid-frontal location in PDFoG+ compared with PDFoG–	Associated with impairments in cognitive control (5th)
		Beta (13–30 Hz) power	Increase in the frontal location in PDFoG+ compared with PDFoG–	Altered preparatory adjustments and motor plan execution through top-down signaling (5th or 3rd)
High beta activity in the subthalamic nucleus and freezing of gait in Parkinson's disease (26)	EEG and deep brain electrode recording during seated position	High-beta (21–35 Hz)	Higher STN activity during the OFF state in PDFoG+ compared with PDFoG–	Associated with interference in the frontal cortico-BG loops, which suggests a predisposition to freeze (2nd)
Attention modulation during motor preparation in parkinsonian freezers: A time-frequency EEG study (105)	Discriminatory attentional dual task, as mediated by oddball paradigm using EEG, for the time preceding gait initiation	Beta ERD	Not present in PDFoG+ compared with PDFoG–	Associated with impairments of the BG, as either hypersynchronized thalamocortical activity or reduced BG involvement (2nd)
		Beta ERS	Prolonged in PDFoG+ compared with PDFoG–	A cortical marker of the high-power beta oscillations in the subthalamic nucleus during FoG (2nd)
Neuromodulation targets pathological not physiological beta bursts during gait in Parkinson's disease (111)	Walking in place and forward walking comparison of DBS frequency-setting effects while recording STN and EEG power coherence	Beta (13–30) burst	Prolonged in PDFoG+ compared with PDFoG–	Reflect beta-band oscillations in the STN, which are representative of FoG (2nd)
Bicycling suppresses abnormal beta synchrony in the parkinsonian basal ganglia (115)	Comparison of subthalamic features OFF medication during bicycling	Beta (13–35 Hz)	Reduced in PDFoG+ compared with PDFoG–	Reduces interference between cortico-BG loops, thus reducing FoG risk (2nd)
		18-Hz power	Increased at movement onset in PDFoG+ compared with PDFoG–	Indicates susceptibility to freezing caused by movement-inhibition throughout the motor network (2nd)
Subthalamic neural entropy is a feature of freezing of gait in freely moving people with Parkinson's disease (30)	STN activity comparison during stepping in place, forward walking, and instrumented walking	Beta (13–30) power	Decreased during stepping in PDFoG+ compared with PDFoG–	Compensatory mechanism adopted to overcome abnormal gait (2nd)
		Sample entropy	Increased during forward walking in PDFoG+ compared with PDFoG–	Increase in error of the processing of sensorimotor information in the subthalamic outflow that results in abnormal gait patterns (1st or 2nd)

*Results and interpretation of studies that compare neural biomarkers of PDFoG+ vs. PDFoG–*.

### The Difference in STN Activity Between PDFoG+ and PDFoG−

When delving into subcortical studies and LFP, an increased high-beta (21–35 Hz) power was observed in the STN during the OFF state in PDFoG+. Levodopa administration was found to effectively reduce the high-beta power (26). In the study on the power coherence between the STN and EEG, beta-burst duration was longer in PDFoG+ compared with PDFoG– during stepping in place and walking forward (111). In the bicycling and walking study, the greatest beta-band decrease was observed during cycling, indicating its potential therapeutic effect by matching the effects of DBS therapy (115). Finally, beta power was decreased during stepping without FoG and was proposed to indicate a compensatory mechanism adopted to overcome abnormal gait in PDFoG+. This study also explored sample entropy and found it to be at its greatest during forward walking. This indicated an error in the processing of subthalamic outflow, resulting in abnormal gait patterns (30). The vast variety of biomarkers and competing results are suggestive of the need for future research to follow similar paradigm and evaluation procedures. For findings to be significant, future research must agree on the way bands are segmented, i.e., beta into high and low bands. Also, to more closely match potential FoG event occurrences, more work must focus on actual lower limb task paradigms and the execution of protocols during ON vs. OFF medication states.

### Difference in the Electrophysiological Activity Between ON and OFF Medication

Of the two studies resulting from the search query, one examined the effects of parkinsonian medication. The results from such explorations can even be used to support some of the hypotheses by evaluating the known effect of the medication and the symptom alleviation provided. For example, high-beta power reduction was shown to be mediated by levodopa administration, resulting in FoG cessation. This high-beta power reduction resulted in similar oscillatory high-beta levels between the PDFoG+ and PDFoG– groups (26). This finding supports the fifth mechanistic hypothesis, which states that FoG events are caused by a malfunction of the frontal cortex-BG networks via the motor and associative STN loops. This study also found high-beta coherence between the STN and the SMA, which supports the association between the hyperdirect pathway and FoG event triggering. However, dopaminergic medications are not effective for all who develop FoG and may even trigger FoG events, as in PDFoG+ who experience FoG even ON-levodopa. Recent studies have shown that dopaminergic medication improves gait speed, stride length, and reduces freezing events but does not improve gait asymmetry and gait arrhythmicity (7, 10, 119). Premovement EEG beta desynchronization has been shown to be reduced in PDFoG+, and this abnormality is at least partially mitigated by dopaminergic stimulation (5, 50, 119). Additionally, cholinergic loss in the pedunculopontine nucleus may play a role in FoG, as it stands at the crossroads between supraspinal and spinal gait centers (50). fMRI studies to understand the effects of dopamine on cortical–subcortical connectivity within PDFoG+ is an active area of research (120) and may be able to distinguish cortical–subcortical connectivity changes between dopamine responsive PDFoG+ and dopamine non-responsive PDFoG+.

## FoG Event Detection

In addition to the application of biomarker research, EEG may also be used to detect and even predict FoG events. From the search query, the earliest works employed four channels of wireless EEG systems for FoG event detection (102). In this work, the wavelet decomposition method was used because of its adaptable and adjustable characteristics that allow for time-frequency localization, multiscale zooming, and multi-rate filtering (102). A three-layer backpropagation neural network was used with the highest classification accuracy of 76.6% while relying only on the P4 channel. The success rate of classification between normal and freezing onset implied that the FoG event could be detected from the neural signature as far back as 5 s before the physical representation. This work was further advanced to incorporate spatial, spectral, and temporal features as well as a k-nearest neighbor classifier to bump classification accuracy to 80% (121), then 87% (101). The electrodes detected to be most sensitive to the transition of freezing, namely P4 and Cz, support the finding that freezing impacts the medial parietal areas, which are in charge of integrating sensory information and visuospatial processing (122). Other FoG event detection studies relied on effective brain connectivity to boost classification accuracy up to 94.8% (103). In this study, findings suggest that hypersynchronization is generated by the frontal region, which is critical for spatial attention, motor intention, cognition, and decision-making processes. These results support the fifth hypothesis underlying FoG, which states that FoG is a consequence of frontal executive dysfunction, where there is a disconnect between the BG and the frontal lobe, resulting in poor performance when trying to achieve multiple tasks (6, 44, 48). Later studies have also focused on the electrode placement, suggesting an optimal montage of two channels, located at C4 and O2 (123). Meanwhile, others have focused on turning freezing detection (TF), which is a subtype of FoG, to improve detection classification from 68.6% (94) to 86.2% (124). The latest of these studies focused on expanding on the feasibility and robustness of FoG detection with EEG by examining data from more subjects, resulting in a sensitivity and specificity of 82.7 and 86.6%, respectively (125). These studies concluded that FoG can be detected and even predicted through the underlying EEG signature with high certainty and a low EEG channel count.

## Author Contributions

CL, ZM, and OB had the idea for the article. JM, SH, and MS performed the literature search. JM and VM drafted the article. All authors critically revised the work.

## Conflict of Interest

CL has received educational grants and honorary from Medtronic, Abbott, and Boston Scientific. The remaining authors declare that the research was conducted in the absence of any commercial or financial relationships that could be construed as a potential conflict of interest.

## Abbreviations

ACC: Anterior Cingulate Cortex
APA: Anticipatory Postural Adjustments
BP: Bereitschaftspotential
BG: Basal Ganglia
COP: Center of Pressure
CPG: Central Pattern Generators
DBS: Deep Brain Stimulation
DCN: Deep Cerebellar Nuclei
dMRI: Diffusion Magnetic Resonance Imaging
EEG: Electroencephalography
EMG: Electromyography
ERD: Event-Related de-Synchronization
ERP: Event-Related Potential
ERS: Event-Related Synchronization
fMRI: functional Magnetic Resonance Imaging
FoG: Freezing of Gait
GIF: Gait Initiation Failure
GPe: Globus Pallidus pars externus
GPi: Globus Pallidus internus
L-dopa: Levodopa
LFP: Local Field Potential
LRP: Lateralized Readiness Potential
MLR: Mesencephalic Locomotor Region
MRCP: Movement-Related Cortical Potential
PD: Parkinson's Disease
PDFoG–: Persons with PD who do not exhibit FoG
PDFoG+: Persons with PD who exhibit FoG
PDnr: PDFoG+ non-responsive to VR feedback
PDr: PDFoG+ responsive to VR feedback
PET: Positron Emission Topography
PMC: Premotor Cortex
PMRF: Pontomedullary Reticular Formation
PPN: Pedunculopontine Nucleus
pre-SMA: pre-Supplementary Motor Area
SMA: Supplementary Motor Area
SNc: Substantia Nigra pars compacta
SNr: Substantia Nigra pars reticulata
STN: Subthalamic Nucleus
TF: Turning Freezing
TUG: Timed Up-and-Go
vMRI: volumetric Magnetic Resonance Imaging
VR: Virtual Reality.
