# Corrigendum: Neural Correlates of Freezing of Gait in Parkinson's Disease: An Electrophysiology Mini-Review

**DOI:** 10.3389/fneur.2022.864942

**Published:** 2022-03-04

**Authors:** J. Sebastian Marquez, S. M. Shafiul Hasan, Masudur R. Siddiquee, Corneliu C. Luca, Virendra R. Mishra, Zoltan Mari, Ou Bai

**Affiliations:** ^1^Department of Electrical and Computer Engineering, Florida International University, Miami, FL, United States; ^2^Department of Neurology, University of Miami Hospital, Miami, FL, United States; ^3^Lou Ruvo Center for Brain Health, Cleveland Clinic, Las Vegas, NV, United States

**Keywords:** Parkinson's disease (PD), freezing of gait (FoG), electrophysiology, cortical, subcortical

In the original article, there was a mistake in Figure 1 as published. In this brain illustration, the marked frontal lobe does not include the precentral gyrus and the SMA is in proximity to the frontal lobe border. Additionally, in Figure 1 parts H1-H4 the somatosensory cortex (dark green) was mistakenly located in the precentral gyrus, instead of the somatomotor cortex. The corrected Figure 1 (made up of the individual illustrations below) has been corrected.

**Figure 1:**
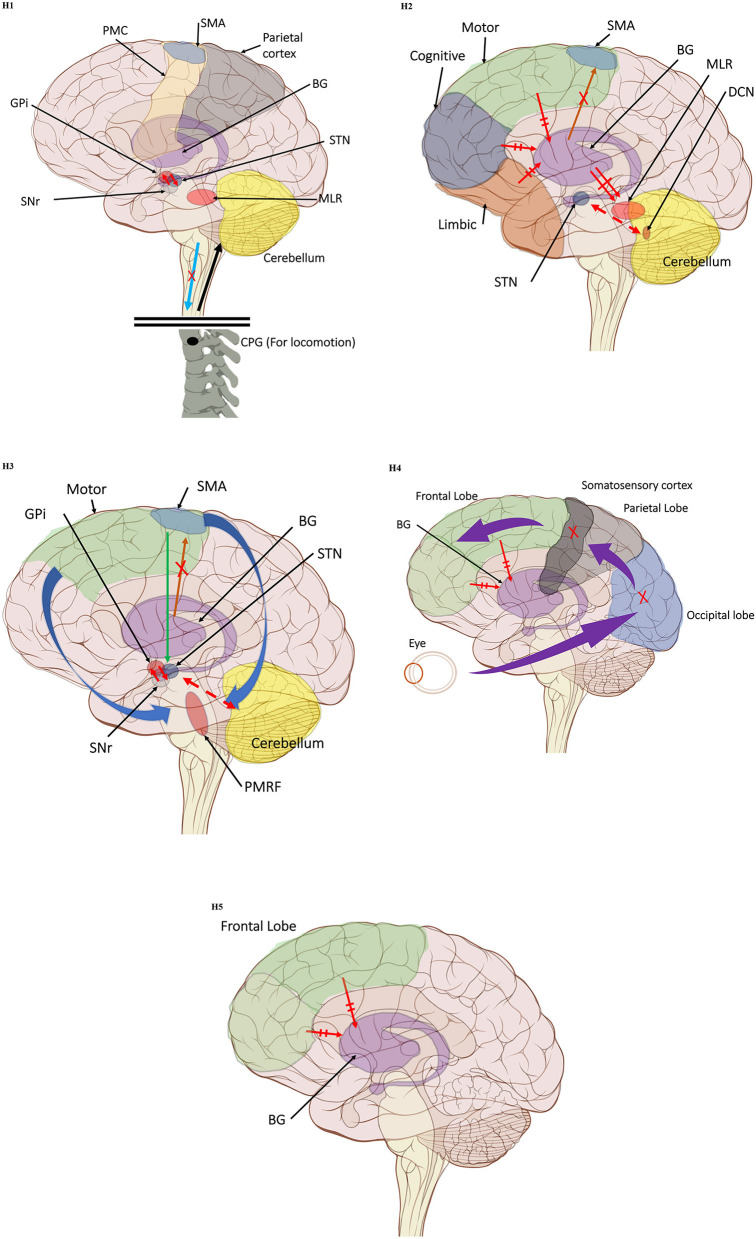
(H1) Abnormal control outputs from central pattern generators. The supraspinal locomotor network (SLN) is composed of the premotor cortex (PMC), supplementary motor area (SMA), parietal cortex, basal ganglia (BG), subthalamic nucleus (STN), mesencephalic locomotor region (MLR), and cerebellum. 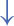 In normal function, the SLN network sends cues for turning, stopping, obstacle maneuvering, and new locomotion goals. X In PD, FoG is caused by the disruption of SLN cues. 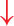 Disruption is caused by inhibition of the globus pallidus internus (GPi)/substantia nigra pars reticulata (SNr) pathway, resulting in decreased rhythmic control. (H2) A disconnect between the basal ganglia and the supplementary motor area or crosstalk to basal ganglia. 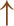 In normal function, BG-SMA sends internal cueing for automatic initiation of previously learned tasks. X In PD, FoG is caused by the disruption of BG-SMA cues. This disconnect leads to the inability to multitask. 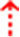 The increased inhibitory output from deep cerebellar nuclei (DCN) further prevents the execution of habitual responses. 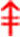 In PD, crosstalk between the input to BG from the cognitive, motor, and limbic cortices may also trigger FoG. 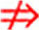 Firing in the output nuclei of the BG inhibits MLR, leading to FoG events. (H3) Knee-trembling and the abnormal coupling between posture and gait. 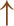 In normal function, BG-SMA sends internal cueing for the automatic initiation of previously learned tasks. Because of the dopamine depletion, executive function is lost. X The BG then fails to convey appropriate anticipatory postural adjustments. 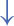 The breakdown of coupling between posture preparation (SMA) and step initiation (motor cortex) might occur at the pontomedullary reticular formation (PMRF), which plays a role in postural control and regular locomotion. 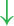 The hyperdirect pathway (SMA-STN) becomes engaged as a result of the coupling breakdown activating the GPi/SNr pathway. 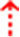 This additionally affects cerebellar automatic gait processing. 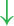 GPi/SNr oscillations may underpin characteristic 3–8 Hz knee-trembling. (H4) A perceptual malfunction and slowing down when passing doorways. 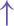 In normal function, the dorsal stream takes visual information to the occipitoparietal stream, where somatosensory signals are then transferred to the frontal lobe for the origination of motor function intent. X In PD, FoG events are caused by dysfunctional dorsal stream processing, which consequently causes inappropriate adaptation of locomotion. (H5) A consequence of frontal executive dysfunction. 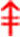 In PD, FoG events are caused by a disconnect between the frontal lobe and the BG. This disconnect results in poor performance of multiple consequent tasks and the characteristically low frontal assessment battery and verbal fluency scores. Highlighted regions and added text and arrows to Medical Illustrations by Patrick Lynch, generated for multimedia teaching projects by the Yale University School of Medicine, Center for Advanced Instructional Media, 1987-2000. Patrick J. Lynch, http://patricklynch.net Creative Commons.

The authors apologize for this error and state that this does not change the scientific conclusions of the article in any way. The original article has been updated.

## Publisher's Note

All claims expressed in this article are solely those of the authors and do not necessarily represent those of their affiliated organizations, or those of the publisher, the editors and the reviewers. Any product that may be evaluated in this article, or claim that may be made by its manufacturer, is not guaranteed or endorsed by the publisher.

